# The Fatty Liver Assessment in Germany (FLAG) cohort study identifies large heterogeneity in NAFLD care

**DOI:** 10.1016/j.jhepr.2020.100168

**Published:** 2020-08-04

**Authors:** Wolf Peter Hofmann, Peter Buggisch, Lisa Schubert, Nektarios Dikopoulos, Jeannette Schwenzer, Marion Muche, Gisela Felten, Renate Heyne, Patrick Ingiliz, Anna Schmidt, Kerstin Stein, Heiner Wedemeyer, Thomas Berg, Johannes Wiegand, Frank Lammert, Stefan Zeuzem, Jörn M. Schattenberg

**Affiliations:** 1 Gastroenterologie am Bayerischen Platz, Berlin, Germany; 2 Association of Gastroenterologists in Private Practice (Berufsverband Niedergelassener Gastroenterologen Deutschlands), Ulm, Germany; 3 IFI Institut für interdisziplinäre Medizin, Hamburg, Germany; 4 Medical Department (Gastroenterology, Infectiology, Rhumatology) Charité Campus Benjamin Franklin, Berlin, Germany; 5 Gastroenterologische Schwerpunktpraxis, Dornstadt, Germany; 6 Bauchzentrum Biesdorf, Berlin, Germany; 7 Gastroenterologische Praxis Herne, Herne, Germany; 8 Leberzentrum am Checkpoint, Berlin, Germany; 9 Zentrum für Infektiologie Prenzlauer Berg, Berlin, Germany; 10 Magen-Darm-Zentrum Wiener Platz, Cologne, Germany; 11 Praxis für Infektiologie und Hepatologie Magdeburg, Magdeburg, Germany; 12 Department of Gastroenterology, Hepatology and Endocrinology, Hannover Medical School, Hannover, Germany; 13 Division of Hepatology, Department of Medicine II, Leipzig University Hospital, Leipzig, Germany; 14 Department of Medicine II, Saarland University Hospital, Homburg, Germany; 15 Department of Internal Medicine, Goethe University Hospital Frankfurt, Frankfurt, Germany; 16 I. Department of Medicine, University Medical Centre of the Johannes Gutenberg-University, Mainz, Germany; 17 Metabolic Liver Research Program, University Medical Centre of the Johannes Gutenberg-University, Mainz, Germany

**Keywords:** NASH, NAFLD, Real world, Metabolic syndrome, Liver fibrosis, Co-morbidities, ALT, alanine aminotransferase, APRI, aspartate-aminotransferase-to-platelet ratio index, AST, aspartate aminotransferase, BMI, body mass index, CAP, controlled attenuation parameter, CVE, cardiovascular event, FAST, FibroScan-AST, FIB-4, fibrosis-4, FLAG, Fatty Liver Assessment in Germany, GLP-1, glucagon-like peptide-1, LSM, liver stiffness measurement, NAFL, non-alcoholic fatty liver, NAFLD, non-alcoholic fatty liver disease, NASH, non-alcoholic steatohepatitis, T2DM, type 2 diabetes mellitus, GGT, gamma-glutamyltransferase

## Abstract

**Background & Aims:**

NAFLD is a growing health concern. The aim of the Fatty Liver Assessment in Germany (FLAG) study was to assess disease burden and provide data on the standard of care from secondary care.

**Methods:**

The FLAG study is an observational real-world study in patients with NAFLD enrolled at 13 centres across Germany. Severity of disease was assessed by non-invasive surrogate scores and data recorded at baseline and 12 months.

**Results:**

In this study, 507 patients (mean age 53 years; 47% women) were enrolled. According to fibrosis-4 index, 64%, 26%, and 10% of the patients had no significant fibrosis, indeterminate stage, and advanced fibrosis, respectively. Patients with advanced fibrosis were older, had higher waist circumferences, and higher aspartate aminotransferase and gamma-glutamyltransferase as well as ferritin levels. The prevalence of obesity, arterial hypertension, and type 2 diabetes increased with fibrosis stages. Standard of care included physical exercise >2 times per week in 17% (no significant fibrosis), 19% (indeterminate), and 6% (advanced fibrosis) of patients. Medication with either vitamin E, silymarin, or ursodeoxycholic acid was reported in 5%. Approximately 25% of the patients received nutritional counselling. According to the FibroScan-AST score, 17% of patients presented with progressive non-alcoholic steatohepatitis (n = 107). On follow-up at year 1 (n = 117), weight loss occurred in 47% of patients, of whom 17% lost more than 5% of body weight. In the weight loss group, alanine aminotransferase activities were reduced by 20%.

**Conclusions:**

This is the first report on NAFLD from a secondary-care real-world cohort in Germany. Every 10th patient presented with advanced fibrosis at baseline. Management consisted of best supportive care and lifestyle recommendations. The data highlight the urgent need for systematic health agenda in NAFLD patients.

**Lay summary:**

FLAG is a real-world cohort study that examined the liver disease burden in secondary and tertiary care. Herein, 10% of patients referred to secondary care for NAFLD exhibited advanced liver disease, whilst 64% had no significant liver scarring. These findings underline the urgent need to define patient referral pathways for suspected liver disease.

## Highlights

• This is the first report on NAFLD from a secondary-care real-world cohort in Germany.
• In this outpatient cohort, diagnostics rely heavily on non-invasive surrogate scores and elastography to stage NAFLD.
• Every 10th patient presents with advanced fibrosis at baseline.
• Management of NAFLD in Germany consists predominantly of general lifestyle recommendations and best supportive care.
• The study underlines the applicability of non-invasive tests to identify patients at risk of advanced fibrosis in secondary care in Germany.

## Graphical abstract

**Figure:**
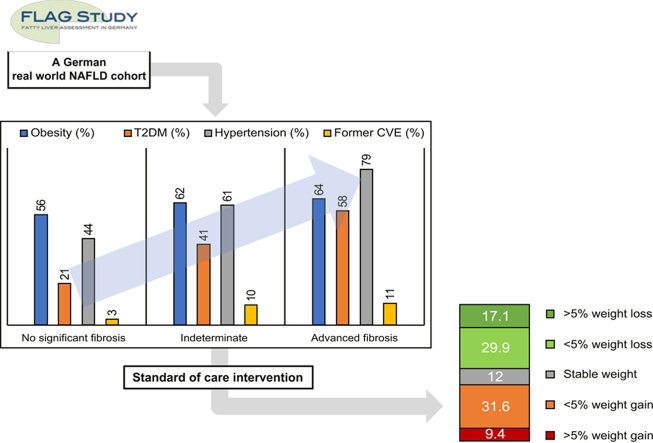

## Introduction

Globally, NAFLD is the most common liver disease with an estimated prevalence of 24%.^1^ NAFLD constitutes a progressive disease spectrum ranging from non-inflammatory steatosis (non-alcoholic fatty liver), hepatitis (NASH), to liver cirrhosis.^2^ More recently the term metabolically associated fatty liver disease has been suggested to provide positive criteria in the definition of the disease spectrum and to overcome limitations related to the role of social, non-abusive alcohol use.^3^ At the individual level, patients are burdened with impaired quality of life^4^ and the risk to develop end-stage liver disease and its sequelae, including hepatic decompensation and hepatocellular carcinoma. At the societal level, the disease generates high economic and healthcare expenditures.^5^ In 2013, end-stage liver disease related to NAFLD was the second most common cause for liver transplantation in the USA.^6^

Currently, several randomised controlled trials are exploring pharmacotherapies for patients with progressive NASH, and a first trial that studied obeticholic acid in comparison with placebo has reported a positive interim analysis showing fibrosis regression after 18 months of treatment.^7^ In regulatory trials, patients are highly selected using liver histology to define disease stage and disease activity, whereas real-world data on the epidemiology of patients with NAFLD are largely unknown. In a German tertiary care cohort using liver histology to define advanced non-cirrhotic fibrosis, the prevalence of F3 fibrosis was 15.6%,^8^ but population-based estimates report only 400,000 F3 cases in Germany.^9^ Thus, the real proportion of NAFLD patients who may benefit most from lifestyle changes and/or upcoming NASH pharmacotherapies is not well defined. Currently, only few national patient real-world registries in Europe or the USA are underway to close these knowledge gaps, and international expert panels request better public health actions, which may include the promotion of referral algorithms, structured lifestyle programmes, awareness campaigns, and national registries.^10^^,^^11^

The Fatty Liver Assessment in Germany (FLAG) study is a prospective observational real-world cohort study performed in secondary and tertiary care. Our aim was to explore the characteristics, disease severity, and patterns of care in patients with NAFLD in Germany referred to secondary care. Additionally, prospective follow-up data at 12 months during standard of care are reported.

## Patients and methods

### Study population and ethical considerations

The FLAG study is an observational real-world cohort study initiated by the Association of Gastroenterologists in Private Practice (Berufsverband Niedergelassener Gastroenterologen Deutschlands) in cooperation with academic medical centres and the German Liver Foundation covering secondary and tertiary healthcare levels. Data collection is performed using an electronic case report form, and data quality is verified by plausibility checks and off-site monitoring. The study protocol was approved by the local ethics committee (Ärztekammer Berlin, Berlin, Germany; 51/16). Written informed consent was obtained from each patient included in the study. The study protocol conforms to the ethical guidelines of the Declaration of Helsinki.

Data were derived from 13 sites across Germany, including office-based practices (9/13) and academic outpatient clinics (4/13). Patient baseline data were recorded between May 2017 and October 2019, resulting in a mean recruitment rate of 17 patients per month.

Inclusion criteria consisted of men and women aged ≥18 years in whom diagnosis of NAFLD was based on:

• hepatic steatosis assessed by ultrasound or pathological controlled attenuation parameter (CAP) measurements;
• availability of clinical, technical, and laboratory data to compute the fibrosis-4 (FIB-4) index, the aspartate-aminotransferase (AST)-to-platelet ratio index (APRI) score, and/or the NAFLD fibrosis score as well as liver stiffness measurements (LSMs);
• data availability to assess components of the metabolic syndrome; and
• alcohol consumption for men <30 g/day and women <20 g/day, respectively.

Patients were excluded if they had evidence of other chronic liver diseases, such as alcoholic liver disease, chronic HBV and HCV infections, autoimmune or cholestatic liver disease, haemochromatosis, alpha1-antitrypsin deficiency, or Wilson disease. Patients who had a history of hepatotoxic medication, such as methotrexate, amiodarone, or long-term NSAID, as well as patients with malignant diseases 12 months before study enrolment were also excluded.

### Routine laboratory testing and assessment of medical history

Laboratory testing comprised a routine hepatology workup, including the liver enzymes AST, alanine aminotransferase (ALT), and gamma-glutamyltransferase (GGT), and blood cell counts, as well as lipid profiles, serum ferritin concentrations, and HbA1c measurements. Chronic liver diseases other than NAFLD were ruled out according to widely used hepatology panels.^12^ The presence of components of the metabolic syndrome in patients were assessed standardized including waist circumference and body mass index (BMI), as well as the presence of arterial hypertension, type 2 diabetes mellitus (T2DM). Additionally, former cardiovascular events (CVEs) were recorded.

### Non-invasive liver fibrosis scoring surrogates

Non-invasive fibrosis scores were used to classify patients into no significant fibrosis, intermediate range, or advanced fibrosis. The FIB-4 index (lower cut-off <1.45; higher cut-off >2.67, or >3.25, respectively), the APRI score (lower cut-off ≤0.50; higher cut-off >1.50), the NAFLD fibrosis score (lower cut-off <−1.455; higher cut-off >0.676), and the FibroScan-AST (FAST) score were computed from available parameters.[13], [14], [15], [16] LSMs (lower cut-off 8.2 kPa; higher cut-off 9.6 kPa according to Eddowes *et al.*^17^) and CAP (dB/m) using the FibroScan® device were available in 251 (50%) and 107 patients (21%), respectively. Supplementary Table S1 gives an overview of the used non-invasive scoring surrogates.

### Prediction of progressive NASH

The identification of patients who may benefit from emerging NASH pharmacotherapies is uncertain when simple non-invasive fibrosis scores, such as the FIB-4 index, are applied. The non-invasive FAST score, which constitutes the parameters LSM (kPa), CAP (dB/m), and AST (U/L), has recently been developed to identify individuals with NASH, significant inflammatory activity, and fibrosis, who may represent candidates for new NASH therapies.^16^ Importantly, the accuracy of non-invasive test depends on the pretest probability of the target lesion, and therefore different cut-offs have been described in the literature for different populations. Recently, an LSM cut-off >9.1 kPa has been proposed to identify patients with liver fibrosis ≥F2,^18^ and in the current analysis both cut-offs are explored in the respective results section.

### Lifestyle factors and intervention assessment

Smoking habits (current smoker or non-smoker) and alcohol consumption were assessed with regard to frequency (never, occasionally, or regularly) and quantified according to daily cut-offs. Patients were asked if they ever received nutritional counselling and about physical exercise habits (never, ≤2 times per week, or >2 times per week).

### Subset of patients with follow-up data

In 117 out of 507 patients (23%) from the entire cohort, follow-up year 1 data were available, including liver enzymes, information on biometrical data, and medical history data. The mean (SD) follow-up time was 12.4 (1.6) months.

### Statistical analyses

Descriptive statistics are used to describe the study population. Differences between groups were tested using the non-parametric chi-square tests for categorical variables and the Kruskal-Wallis test or Wilcoxon test for continuous variables. In addition, *p* values <0.05 were considered statistically significant. Analyses were carried out using SPSS software (SPSS Inc., Chicago, IL, USA).

## Results

### Study population

The present analysis includes 507 patients with a mean age 53 years; 268 were men (53%) and 239 were women (47%). More than two-thirds of the patients were recruited at office-based practices (n = 360; 71%) and the remaining 147 (29%) patients were recruited at academic sites. Patients reported predominantly Caucasian ethnicity (89%). Twenty-three patients were anti-hepatitis B core antibody positive, and 6 patients had anti-HCV antibodies without evidence of replicative viral hepatitis (HCV RNA negative). Sixty patients (11%) had low titres of anti-nuclear antibodies, but no evidence of autoimmune hepatitis (including normal IgG concentrations).

### Non-invasive fibrosis scores

By the use of non-commercial and easily accessible scoring surrogates, including FIB-4 index, NAFLD fibrosis score, and APRI score, the cohort was grouped into patients with no significant fibrosis, indeterminate stage, and advanced fibrosis (Table 1). Because of incomplete data available for the parameter albumin, NAFLD fibrosis score was calculated in only 366 out of 507 patients. More patients from academic sites had complete parameters to calculate the NAFLD fibrosis score (Supplementary data). Furthermore, LSMs were only available in 251 out of 507 patients. However, LSMs were equally available from office-based practices and academic sites, respectively (Supplementary data). We computed the FIB-4 index with the original cut-off >3.25 as well as with the recently described cut-off >2.67 for advanced fibrosis (Table 1), which has been shown with increased sensitivity for the detection of advanced fibrosis in a recent meta-analysis.^15^ By using the latter cut-off, 64%, 26%, and 10% of the patients were grouped into no significant fibrosis, indeterminate, and advanced fibrosis stages, respectively, and this cut-off was used for subsequent analyses.^15^^,^^19^ With respect to FIB-4 and LSM-derived fibrosis stage, there were no significant differences between patients recruited at office-based practices and academic sites (Supplementary data).

**Table 1:** Fibrosis stage according to different non-invasive scoring surrogates.

	No significant fibrosis, n (%)	Indeterminate, n (%)	Advanced fibrosis, n (%)
FIB-4 (n = 507; cut-off <1.45 and >3.25)	324 (64)	143 (28)	40 (8)
FIB-4 (n = 507; cut-off <1.45 and >2.67)	324 (64)	130 (26)	53 (10)
NAFLD fibrosis score (n = 366)	134 (37)	110 (30)	122 (33)
APRI score (n = 507)	479 (94)	25 (5)	3 (1)
LSM (n = 251)	166 (66)	17 (7)	68 (27)

LSM cut-offs for indeterminate stage and advanced fibrosis were 8.2 and 9.6 kPa, respectively, according to Eddowes *et al*.^17^ NAFLD fibrosis score cut-offs and APRI score cut-offs were according to the original publication^13^^,^^14^; see also Supplementary data. Chi-square test was used for categorical variables, Kruskal-Wallis test or Wilcoxon test for continuous variables.

APRI, aspartate-aminotransferase-to-platelet ratio index; FIB-4 index, fibrosis-4 index^19^^,^^20^; LSM, liver stiffness measurement.

### Clinical characteristics

Table 2 summarises the baseline characteristics and demographics according to fibrosis stages. There were several significant differences within the 3 groups. Patients with advanced fibrosis were older; had greater waist circumferences; and showed highest levels of AST, GGT, and ferritin. The frequencies of co-morbidities, including obesity, arterial hypertension, and T2DM, were highest in patients with advanced fibrosis. Former CVEs were more frequently observed in indeterminate and advanced fibrosis stage compared with the remaining no significant fibrosis patients (Table 2). Whilst LSM differed significantly between groups, CAP measurements did not show differences according to fibrosis stages.

**Table 2:** Baseline characteristics and demographics according to fibrosis stage (FIB-4 cut-off >2.67 for advanced fibrosis).

	No significant fibrosis (n = 324)	Indeterminate (n = 130)	Advanced fibrosis (n = 53)	*p* value
Age (years)	48 (13)	61 (9)	65 (7)	<0.001
Men (%)	56	47	47	0.121
BMI (kg/m^2^)	30 (5)	31 (6)	31 (5)	0.329
Waist circumference (cm)	103 (13)	108 (15)	108 (12)	0.007
ALT (U/L)	65 (41)	62 (38)	62 (29)	0.595
AST (U/L)	39 (19)	51 (28)	66 (29)	<0.001
GGT (U/L)	89 (75)	122 (110)	231 (176)	<0.001
Platelets (g/dl)	267 (63)	207 (51)	133 (44)	<0.001
HbA1c (mg %)	6.64 (7.89)	7.01 (8.10)	6.47 (1.32)	<0.001
Ferritin (mg/dl)	223 (181)	305 (287)	352 (285)	0.002
LSM (kPa)	7.5 (4.6)	10.7 (10.7)	24.1 (19.1)	<0.001
CAP (dB/m)	314 (45)	297 (58)	315 (50)	0.202
Obesity (%)	56	62	64	0.302
T2DM (%)	21	41	58	<0.001
Hypertension (%)	44	61	79	<0.001
Former CVE (%)	3	10	11	0.001

Data are shown as mean (SD) unless indicated otherwise. Obesity was defined as BMI ≥30 kg/m^2^.

ALT, alanine aminotransferase; AST, aspartate aminotransferase; BMI, body mass index; CAP, controlled attenuation parameter; CVE, cardiovascular event; GGT, gamma-glutamyltransferase; LSM, liver stiffness measurement; T2DM, type 2 diabetes mellitus (chi-square test for categorial variables, Kruskal-Wallis test or Wilcoxon test for continuous variables).

### Lifestyle factors and interventions

In the present cohort, lifestyle factors and interventions were assessed semi-quantitatively. Table 3 shows smoking habits, alcohol consumption, frequencies of physical activity, and the number of patients who received nutritional counselling. There were no obvious differences within the 3 groups regarding smoking habits and alcohol consumption. In addition, 31–38% of the patients did not drink alcohol, approximately half of the patients reported to drink occasionally, and 11–15% of the patients reported to drink regularly. According to the study protocol, patients were not included in the study if alcohol consumption exceeded 30 g/day for men and 20 g/day for women. Approximately half of the patients in all groups reported that they did not practice any exercise. Moreover, 27–37% reported ≤2 units of physical exercise per week. The widely recommended >2 units per week of physical activity was reported in 17%, 19%, and 6% of patients with no significant, intermediate, and advanced fibrosis, respectively. Approximately 25% of the patients had never received nutritional counselling (Table 3).

**Table 3:** Lifestyle factors and interventions according to fibrosis stage (FIB-4 cut-off >2.67 for advanced fibrosis).

	No significant fibrosis (n = 324)	Indeterminate (n = 130)	Advanced fibrosis (n = 53)
Current smoker (%)	23	12	16
Alcohol consumption (%)			
No alcohol	31	35	38
Occasionally	58	50	50
Regularly	11	15	12
Physical exercise (%)			
No exercise	48	54	57
≤2 times a week	35	27	37
>2 times a week	17	19	6
Nutritional counselling (%)	25	23	27

FIB-4, fibrosis-4.

### Potentially NAFLD-modifying medications

Although there is no approved pharmacotherapy for NAFLD patients available today and guidelines do not conclusively recommend any therapeutic intervention besides weight loss, diet, and physical exercise,^21^ several potentially NAFLD-modifying medications might be used in different clinical settings.^22^ The frequencies of medications, including glucagon-like peptide-1 (GLP-1) agonists, statins, acetylsalicylic acid, and ursodeoxycholic acid, as well as the nutritional supplements vitamins D and E and the herbal compound silymarin are shown in Table 4. Data are presented according to the presence or absence of T2DM for 2 reasons:

• the oral antidiabetic GLP-1 agonist liraglutide has shown antifibrotic effects in NAFLD patients in phase II randomised controlled trials and is available as a weight-lowering medication in non-diabetic patients^23^; and
• vitamin E has been studied together with pioglitazone in an early randomised controlled trial of histologically proven non-diabetic NASH patients.^24^

**Table 4:** Frequencies of relevant co-medications.

	Diabetes mellitus (n = 153), n (%)	No diabetes mellitus (n = 354), n (%)
GLP-1 agonists	6 (3.9)	1 (0.3)
Statins	59 (38.6)	40 (11.5)
Acetylsalicylic acid	31 (20.3)	22 (6.3)
Vitamin E	1 (0.7)	4 (1.2)
Vitamin D	31 (20.3)	50 (14.5)
Silymarin (milk thistle)	1 (0.7)	11 (3.2)
Ursodeoxycholic acid	4 (2.6)	5 (1.4)
Metformin	88 (57)	–
Gliptin	25 (16)	–
Insulin	42 (27)	–

GLP-1, glucagon-like peptide-1.

Generally, the use of potentially NAFLD-modifying medications was low. Only 6 out of 153 patients with T2DM and 1 out of 354 patients without diabetes were treated with GLP-1 agonists in our cohort. Vitamin E supplementation was recorded in 5 patients (1/5 in the T2DM group). Statins, acetylsalicylic acid, ursodeoxycholic acid, and vitamin D were more frequently used in the T2DM group. Silymarin was prescribed more frequently in patients without diabetes (Table 4).

### Prediction of progressive NASH and implications for future treatments

In 107 and 251 patients from our cohort, the FAST score and the >9.1 kPa cut-off, respectively, were computed according to the availability of included parameters (Table 5). Using the higher cut-off >0.67 (‘rule in NASH’) of the FAST score, 16.8% of the population was identified as having progressive NASH. More than half of the patients of whom the FAST score was available were recruited at office-based practices (60 out of 107 patients). By computing the proportion of patients having LSM >9.1 kPa as indicator of significant fibrosis, 29% of the population could be considered as population to treat. Table 5 also shows proportions for patient groups according to FIB-4 fibrosis stages.

**Table 5:** Prediction of progressive NASH and population to treat.

	All patients (%)	Different fibrosis stage groups (FIB-4 cut-off >2.67 for advanced fibrosis)		
		No significant fibrosis (%)	Indeterminate stage (%)	Advanced fibrosis (%)
FAST score (n = 107)				
Rule out NASH	48.6	57.6	40.0	18.2
Grey zone	34.6	36.4	36.7	18.2
Rule in NASH	16.8	6.1	23.3	63.6
LSM >9.1 kPA (n = 251)				
Population to treat (≥F2)	29.3	20.1	30.0	84.0

AST, aspartate aminotransferase; FAST score, FibroScan-AST score with a lower cut-off <0.35 (rule out NASH) and higher cut-off >0.67 (rule in NASH) according to Newsome *et al.*^16^; FIB-4, fibrosis-4; LSM, liver stiffness measurement >9.1 kPa cut-off for ≥F2 fibrosis according to Serra-Burriel *et al.*^18^; NASH, non-alcoholic steatohepatitis.

### Subset of the cohort who completed follow-up year 1 visit

**Fig. 1:**
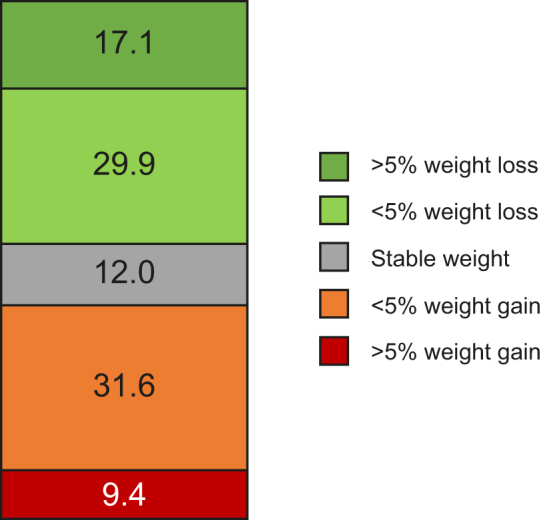
Weight changes in kilograms body weight represented as 5% increments, baseline visit *vs.* follow-up visit, mean (SD) follow-up time was 12.4 (1.6) months, data available in 117 patients of the FLAG cohort. Boxes represent frequencies of weight changes in percent. FLAG, Fatty Liver Assessment in Germany.

**Fig. 2:**
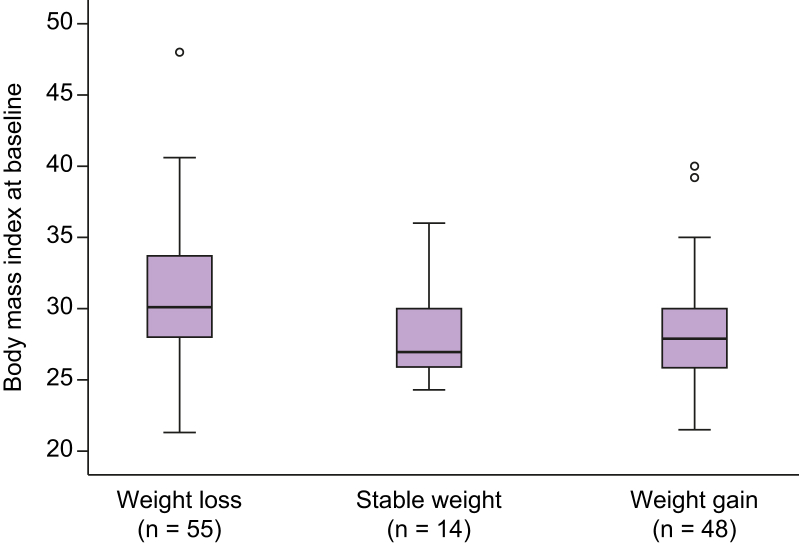
Body mass index (mean; SD; 95% CI) at baseline according to weight change over time (baseline *vs.* follow-up; mean [SD] 12.4 [1.6] months) in 117 patients of the FLAG cohort. Differences within groups: *p* = 0.009 (Kruskal-Wallis test). FLAG, Fatty Liver Assessment in Germany.

**Fig. 3:**
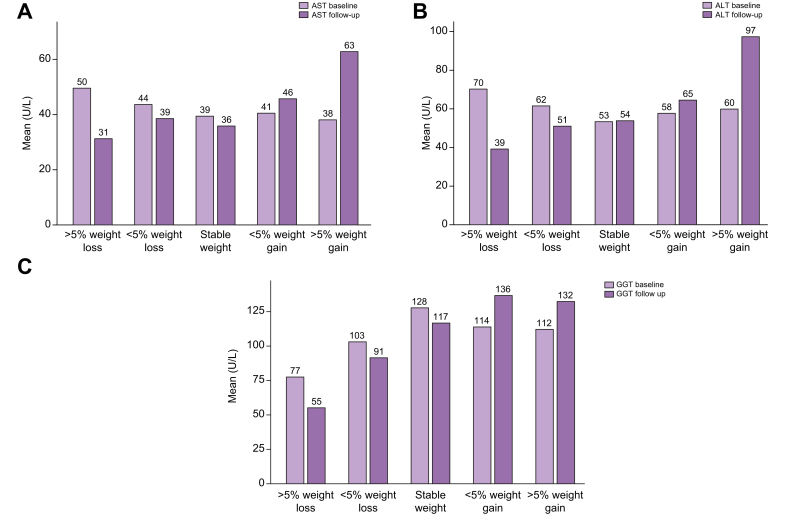
Changes in liver function tests in relation to body weight over 12 months. (A) AST, (B) ALT, and (C) GGT. AST, ALT, and GGT all mean (U/L) at baseline and during follow-up according to weight changes in kilograms body weight represented as 5% increments. Mean (SD) follow-up time was 12.4 (1.6) months. Data were available in 117 patients from the FLAG cohort. ALT, alanine aminotransferase; AST, aspartate aminotransferase; FLAG, Fatty Liver Assessment in Germany; GGT, gamma-glutamyltransferase.

At the time point of the present data analysis, 117 out of 507 patients (23%) completed the year 1 follow-up visit with a mean of 12.4 ± 1.6 months after the baseline visit. Most patients who had a follow-up visit were recruited at office-based practices (104 out of 117). Body weight, BMI, waist circumference, liver function tests, as well as medical history were recorded. The body weight remained stable, increased or decreased in 14 (12%), 48 (41%), and 55 (47%) patients, respectively. Figure 1 shows weight changes in kilograms body weight as 5% increments. The baseline mean BMI differed significantly between those patients who remained stable, gained weight, or lost weight over the 12-month period (Fig. 2). Liver enzyme activity (AST, ALT, and GGT) changed over time in those who gained or lost weight. In those patients who lost weight, reductions of AST, ALT, and GGT activities were 11%, 20%, and 14%, respectively. Weight gainers showed increases of AST, ALT, and GGT levels by 24%, 26%, and 22%, respectively. Figure 3 illustrates mean liver enzyme activities at baseline and during follow-up according to 5% weight change increments. There were only few new CVEs or co-morbidities in our relatively small follow-up subgroup (data not shown).

## Discussion

NAFLD affects 24% of the European population, and its wide disease spectrum ranges from benign hepatic steatosis to progressive NASH, which is associated with a high risk for the development of cirrhosis and its sequelae, including hepatic decompensation and hepatocellular carcinoma.^25^ Although first approved NASH pharmacotherapies are anticipated in the near future,^26^ the identification of patients who benefit most from pharmacotherapy remains challenging, and lifestyle changes, including diet, weight loss, and exercise, are still the recommended interventions today.^21^ Management strategies of NAFLD patients have been proposed in national and international guidelines; however, real-world epidemiological data mainly derive from practice surveys, expert consensus statements, structured interviews, and retrospective chart reviews.^22^^,^^27^ Here, we present first data from the FLAG real-world cohort study with a broad spectrum of 507 NAFLD patients included that were characterised by non-invasive surrogate scores.

Liver histology has been used to define primary treatment endpoints in regulatory trials, but will not be available in the majority of affected patients. Therefore, validated surrogate scores of advanced fibrosis are used to stage the disease severity. The most accurate non-invasive identification of NAFLD patients with advanced fibrosis has been studied extensively, and in addition to LSM, NAFLD fibrosis score and the FIB-4 index perform best^15^^,^^18^; however, cut-off values to rule out significant fibrosis may vary between different chronic liver diseases and populations. By the use of the recently proposed FIB-4 cut-off (>2.67 for advanced fibrosis), 64%, 26%, and 10% of our cohort were classified to have no significant fibrosis, an indeterminate stage, or advanced fibrosis, respectively. As our cohort is relatively small and patient age did not show a broad range, we did not adjust the FIB-4 index calculations by age as previously proposed.^28^ In a recent mathematical model, the overall prevalence of NAFLD in Germany has been estimated to be 23%, including only 3.3% of F3 and F4 patients (600,000 cases).^9^ By contrast, in an academic care cohort, F3 patients alone represented 16% of all cases.^8^ As the referral rate of NAFLD patients from primary care physicians to specialists is largely unknown in Germany, our cohort may be biased towards more advanced disease compared with the estimates from mathematical models, but may still represent a broader NAFLD spectrum compared with tertiary care settings. In other countries, the referral rate from primary care differs largely and has been reported to range from 10% to 80%.^27^^,^^29^ The NAFLD fibrosis score, which includes serum albumin, was computed only in 366/507 cases and seems thus to be less useful in secondary care in Germany. Interestingly, the proportion of patients with advanced fibrosis according to the NAFLD fibrosis score was higher in patients from academic sites compared with office-based practices, whereas no such difference was found when FIB-4 or LSMs were analysed. This difference is most likely explained by an almost doubled prevalence of patients with T2DM amongst academic sites, and raises questions about the use of NAFLD fibrosis score in heterogeneous patient cohorts. LSMs (FibroScan device) were recorded in approximately half of the patients included, although currently most healthcare providers are not reimbursed for occurring costs.

In line with previous studies, baseline characteristics and demographics showed the presence of obesity in 56–64% across all fibrosis stages. Patients with indeterminate stage and advanced fibrosis were older and had higher waist circumferences—a surrogate for abdominal obesity—when compared with those without significant fibrosis. Also, co-morbidities, including arterial hypertension, T2DM, and CVE, were more frequent in advanced fibrosis stages, consistent with previous studies from other parts of the world.^30^ Laboratory testing showed that simple parameters, including GGT and ferritin, were higher in the more advanced population, and earlier studies indicated serum ferritin to be an independent predictor of advanced fibrosis in biopsy-proven NAFLD.^31^

Smoking and alcohol consumption were recorded semi-quantitatively. Whilst this does not allow for a differential exploration of the relative contribution of small amounts of alcohol in patients with metabolic liver disease, the current cohort does not include patients with relevant alcoholic liver injury. Importantly, these lifestyle factors apparently do not differ between the different fibrosis stages. Looking at the standard of care, the frequencies of exercise amongst patients were low with approximately half of the patients reporting a sedentary lifestyle. The guideline recommendation to exercise at least twice weekly was followed least by patients with advanced fibrosis (6%). Furthermore, only 25% of all patients ever received nutritional counselling. These data underline the need of a health agenda supporting effective lifestyle changes in secondary care.[32], [33], [34]

The use of potentially NAFLD-modifying medications has been reported based on surveys amongst gastroenterologists and hepatologists from France, Romania, and the USA.^22^ In France, specialists reported to add pharmacotherapy to lifestyle changes in up to 28% of the cases. Here metformin, ursodeoxycholic acid, glitazones, and vitamin E were prescribed for NAFLD.^29^ The current real-world data from this German cohort highlight a relatively low frequency of pharmacotherapy that has historically been propagated for NAFLD. The use of vitamin E, silymarin, and ursodeoxycholic acid ranged from 0.7% to 3.2%. In particular, vitamin E, which is used more frequently in other countries, including the USA^35^ is not encouraged by the German NAFLD guideline.^21^ The subgroup of patients with T2DM showed a higher prescription rate of GLP-1 agonists, statins, vitamin D, and acetylsalicylic acid compared with those without T2DM.

In the near future, the approval of novel NASH therapies is anticipated. However, the proportion of NAFLD patients who benefit most from those therapies is still on debate and is being studied in long-term randomised controlled phase III trials.^36^ Currently, accepted endpoints for conditional drug approval include resolution of NASH without worsening of fibrosis and/or improvement of fibrosis without worsening of NASH by evaluation of paired liver histologies. As the acceptance of liver biopsies is low both in physicians and patients, novel non-invasive scores, such as the non-commercial FAST score, have been proposed to identify patients who are at greater risk for disease progression and who might be candidates for clinical trials and approved NASH therapies.^16^ The FAST score constitutes the parameters LSM (kPa), CAP (dB/m), and AST (U/L), and aims to ‘rule in NASH’ or to ‘rule out NASH’. In the subset with available FAST score, 16.8%, 34.6%, and 48.6% were classified as ‘rule in NASH’, grey zone, and ‘rule out NASH’, respectively.

The present cohort study has been designed to assess demographic data in a real-world setting over time. Here, we present results from the first 117 patients who completed the follow-up year 1 visit. The body weight remained stable, increased, or decreased in 12%, 41%, and 47% of our patients, respectively. Interestingly, those patients who lost weight had higher baseline BMI values than those who remained stable or gained weight. Previous interventional studies with an intensive lifestyle change observed a weight loss >5% compared with baseline in 30% of the cases.^32^ Similarly, in a study with an internet-based approach for lifestyle changes, the weight loss of 10% was achieved in 15–20% of the patients.^37^ In our non-interventional cohort, 17% of patients achieved a >5% weight reduction at 1 year. However, approximately the same number of patients gained weight. In the group that reduced body weight by more than 5% at 1 year, AST, ALT, and GGT enzyme activities decreased in the mean by 11%, 20%, and 14%, respectively. Data from a recent weight loss trial using the GLP-1 analogue semaglutide showed ALT reductions between 6% and 21% in patients with diabetes and/or obesity.^38^

The results of our study highlight the urgent need to define referral pathways for patients with NAFLD that are seen in primary care in Germany. However, a major weakness of our real-world cohort is the lack of histology, and thus the inability to define disease severity beyond there herein used non-invasive surrogates. Nevertheless, we believe that implementation of readily available scores, such as the FIB-4 index, will facilitate decision-making in primary care when patients are selected for referral to specialists. A recent study from the UK showed that a simple pathway reduces the number of referrals to secondary care and improves the detection rate of patients with advanced fibrosis and cirrhosis.^39^ In our study, we observed a relatively large number of patients without signs of significant fibrosis that were equally distributed in office-based practices and academic centres. These data suggest that the implementation of referral pathways in primary care could also reduce the economic burden of NAFLD in Germany by reducing the number of tests in patients without significant disease.

In summary, we report the characteristics and demographics from an observational NAFLD cohort in Germany managed in secondary and tertiary care. Approximately 10% of patients present with advanced fibrosis, and every 6th patient could be eligible for pharmacotherapy once approved. The low uptake and frequency of lifestyle interventions demonstrate that public health agenda and prevention efforts dedicated to patients with NAFLD are urgently required.^11^

## Financial support

This work was in part funded by grants of the Association of Gastroenterologists in Private Practice (Berufsverband Niedergelassener Gastroenterologen Deutschlands) and the German Liver Foundation to the Fatty Liver Assessment in Germany study group.

## Authors' contributions

Conceptualisation: WPH, PB, PI, HW, TB, FL, SZ, JMS. Funding acquisition: WPH, PI, PB. Investigation: ND, JS, MM, GF, RH, PI, AS, KS. Data curation: WPH. Formal analysis: WPH, LS, JMS. Project administration: LS. Supervision: TB, SZ, HW, JMS. Writing of original draft: WPH. Writing/review/editing: JW, JMS.

## Conflicts of interest

JMS has acted as consultant to GENFIT, Gilead Sciences, Intercept Pharmaceuticals, IQVIA, Madrigal, Roche, Siemens Healthineers, Novartis and Pfizer, and has received research funding from Gilead Sciences. WPH has acted as consultant to Gilead Sciences, Intercept Pharmaceuticals, Takeda, and Allergan, and has received speaker honoraria from Gilead Sciences, AbbVie, Intercept Pharmaceuticals, and Allergan.

Please refer to the accompanying ICMJE disclosure forms for further details.

## Supplementary data

Supplementary information.pdf

CTAT table.pdf
