# Supplementary material for: The Fatty Liver Assessment in Germany (FLAG) cohort study identifies large heterogeneity in NAFLD care
Source: JHEP Rep. 2020 Aug 4;2(6):100168. doi: 10.1016/j.jhepr.2020.100168 (PMC7490844; doi:10.1016/j.jhepr.2020.100168)
Supplement: Supplementary information.pdf [file mmc1.pdf]

The Fatty Liver Assessment in Germany (FLAG) cohort study identifies large heterogeneity in NAFLD care

Wolf Peter Hofmann, Peter Buggisch, Lisa Schubert, Nektarios Dikopoulos, Jeannette Schwenzer, Marion Muche, Gisela Felten, Renate Heyne, Patrick Ingiliz, Anna Schmidt, Kerstin Stein, Heiner Wedemeyer, Thomas Berg, Johannes Wiegand, Frank Lammert, Stefan Zeuzem, Jörn M. Schattenberg

Table of contents

Table S1……………………………………………………………………………………2

Table S2……………………………………………………………………………………3

Fig. S1………………………………………………………………………………………4

Table S1. Overview of the investigated non-invasive fibrosis markers

Test	Calculation method	Upper cutoff	Lower cutoff	Reference
NFS	-1.675+0.037xage(years)+0.094xBMI(kg/m2)+ 1.13xdiabetes (yes=1)+0.99xAST/ALTratio- 0.013xplatelet(x10^9/l)-0.66xalbumin(g/dl)	0.676 irrespective of age	-1.455	[1]
FIB-4 score	Age x AST/platelet count (x10^9/l)x radical(ALT)	2.67<br>3.25<br>3.25	1.3<br>1.3<br>1.45	[2]<br>[3]<br>[4]
APRI	[(AST/ULN AST) / Platelets (x10^9/l)] x100	1.5	0.5	[5]

[1] Angulo P, Hui JM, Marchesini G, Bugianesi E, George J, Farrell GC, et al. The NAFLD fibrosis score: a noninvasive system that identifies liver fibrosis in patients with NAFLD. Hepatology 2007;45:846-854.

[2] Shah AG, Lydecker A, Murray K, Tetri BN, Contos MJ, Sanyal AJ, et al. Comparison of noninvasive markers of fibrosis in patients with nonalcoholic fatty liver disease. Clinical gastroenterology and hepatology : the official clinical practice journal of the American Gastroenterological Association 2009;7:1104-1112.

[3] McPherson S, Stewart SF, Henderson E, Burt AD, Day CP. Simple non-invasive fibrosis scoring systems can reliably exclude advanced fibrosis in patients with non-alcoholic fatty liver disease. Gut 2010;59:1265-1269.

[4] Sterling RK, Lissen E, Clumeck N, Sola R, Correa MC, Montaner J, et al. Development of a simple noninvasive index to predict significant fibrosis in patients with HIV/HCV coinfection. Hepatology 2006;43:1317-1325.

[5] Wai CT, Greenson JK, Fontana RJ, Kalbfleisch JD, Marrero JA, Conjeevaram HS, et al. A simple noninvasive index can predict both significant fibrosis and cirrhosis in patients with chronic hepatitis C. Hepatology 2003;38:518-526.

Table S2. Baseline characteristics and demographics according to heath care level

	office-based practice (n=360)	academic site (n=147)
**gender (men)**	191 (53%)	78 (53%)
**age (years)**	52.6 ± 13.8	53.7 ± 13.7
**BMI**	30.6 ± 5.2	31.6 ± 5.5
**T2DM (%)**	25%	45%
**Arterial hypertension**	49%	61%
**Former CVE**	4%	11%
**LSM (kPa, n=251))**	9.7 ± 10.1	10.6 ± 10.4
**no significant fibrosis (<8.2kPA)**	117 (66%)	49 (65%)
**indeterminate (8.2 – 9.6kPA)**	12 (7%)	5 (7%)
**advanced fibrosis (>9.6kPA)**	47 (27%)	21 (28%)
**FIB-4 (upper cut off >2.67, n=507)**		
**no significant fibrosis**	234 (65%)	90 (61%)
**indeterminate**	89 (25%)	41 (28%)
**advanced fibrosis**	37 (10%)	16 (11%)
**NAFLD fibrosis score**		
**no significant fibrosis**	123 (53%)	11 (8%)
**indeterminate**	66 (28%)	44 (33%)
**advanced fibrosis**	44 (19%)	78 (59%)

Data shown as mean unless indicated otherwise. LSM, liver stiffness measurement; T2DM, type 2 diabetes mellitus; CVE, cardiovascular event. LSM were available in 176 and 75 patients from office-based practices and academic sites, respectively. The NAFLD fibrosis score was available in 233 and 133 from office-based practices and academic sites, respectively.

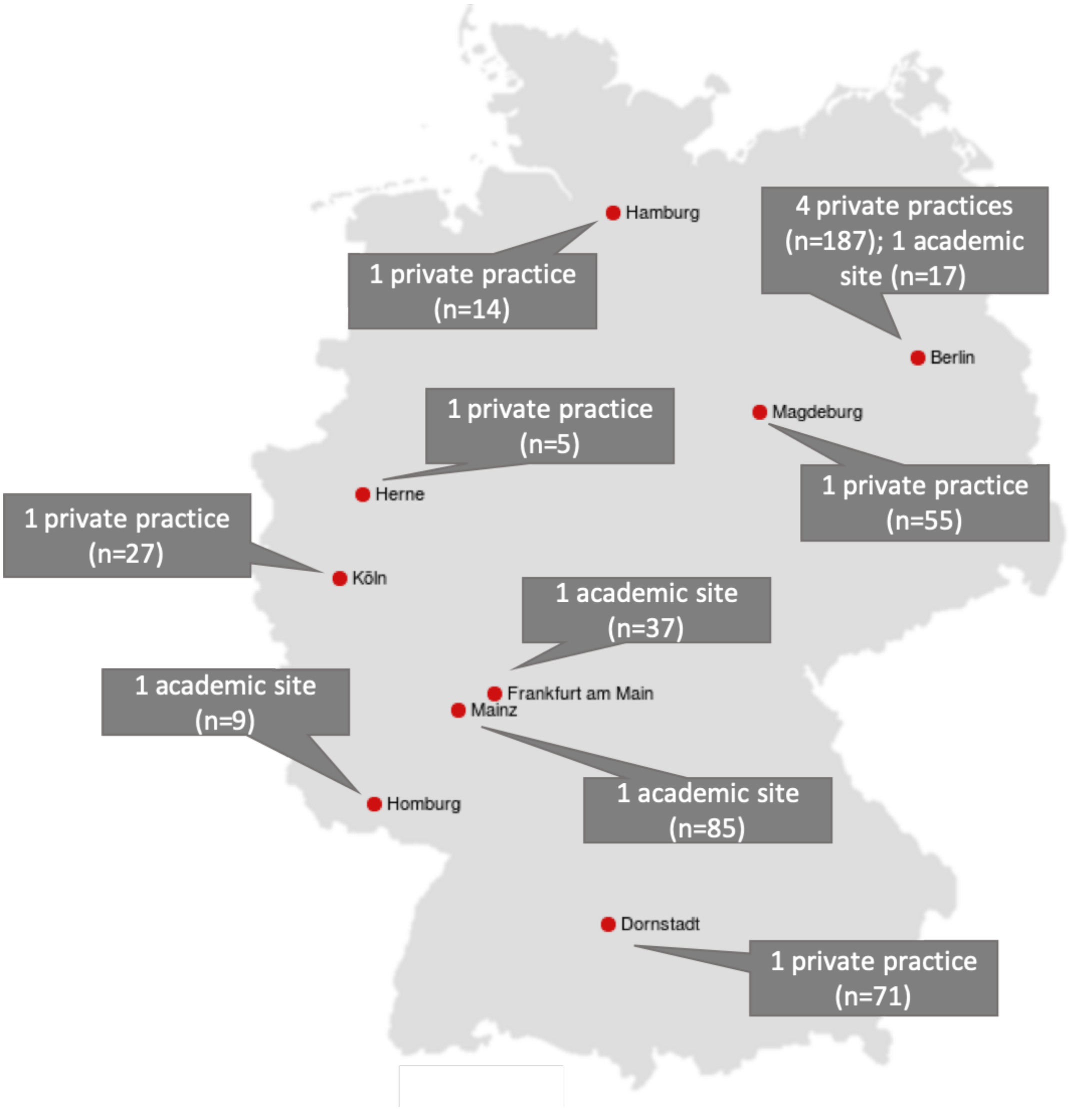

Fig. S1. Shows sites across Germany and gives information about secondary (private practice) and tertiary (academic site) health care level as well as the total number of patients included for the present analysis per site.
